# A demographic and evolutionary analysis of maternal effect senescence

**DOI:** 10.1073/pnas.1919988117

**Published:** 2020-06-29

**Authors:** Christina M. Hernández, Silke F. van Daalen, Hal Caswell, Michael G. Neubert, Kristin E. Gribble

**Affiliations:** ^a^Biology Department, Woods Hole Oceanographic Institution, Woods Hole, MA 02543;; ^b^Institute for Biodiversity and Ecosystem Dynamics, University of Amsterdam, 1090 GE Amsterdam, The Netherlands;; ^c^Josephine Bay Paul Center for Comparative Molecular Biology and Evolution, Marine Biological Laboratory, Woods Hole, MA 02543

**Keywords:** aging, demography, fitness, maternal effects, selection gradients

## Abstract

Maternal effect senescence—a decline in offspring survival or fertility with maternal age—has been demonstrated in many taxa, including humans. Despite decades of phenotypic studies, questions remain about how maternal effect senescence impacts evolutionary fitness. To understand the influence of maternal effect senescence on population dynamics, fitness, and selection, we developed matrix population models in which individuals are jointly classified by age and maternal age. We fit these models to data from individual-based culture experiments on the aquatic invertebrate, *Brachionus manjavacas* (Rotifera). By comparing models with and without maternal effects, we found that maternal effect senescence significantly reduces fitness for *B. manjavacas* and that this decrease arises primarily through reduced fertility, particularly at maternal ages corresponding to peak reproductive output. We also used the models to estimate selection gradients, which measure the strength of selection, in both high growth rate (laboratory) and two simulated low growth rate environments. In all environments, selection gradients on survival and fertility decrease with increasing age. They also decrease with increasing maternal age for late maternal ages, implying that maternal effect senescence can evolve through the same process as in Hamilton’s theory of the evolution of age-related senescence. The models we developed are widely applicable to evaluate the fitness consequences of maternal effect senescence across species with diverse aging and fertility schedule phenotypes.

## Significance

Maternal effect senescence, the decline in offspring quality with increasing maternal age, is common in animals despite its negative impact on fitness. To understand how maternal effect senescence might evolve, we built matrix population models to calculate selection gradients on survival and fertility as functions of maternal age. We estimated the model’s parameters with data from an aquatic invertebrate. The strength of selection eventually declines with age and maternal age, implying that maternal effect senescence could be favored by selection and evolve in the same way as senescence. Our framework can be applied to investigate maternal effect senescence in organisms with diverse life histories and unifies the demographic approaches to age-related and maternal effect senescence.

In many species, survival and reproduction decrease with advancing age, a process known as “senescence.” The evolution of senescence is a long-standing problem in life history theory (1, 2) and has been studied extensively in the laboratory, with mathematical models, and in the field (e.g., refs. 3–6). The evolution of senescence is explained by the age-specific patterns of the strength of selection, measured as selection gradients, the derivatives of the population growth rate with respect to a given trait value. Hamilton (2) showed that age-specific selection gradients on mortality and fertility decrease with age. Thus, traits expressed early in life have a larger impact on fitness than those expressed later. As a result, selection will favor traits that lead to negative effects on survival and fertility at older ages if there are even small beneficial effects in youth.

“Maternal effect senescence” is defined as the reduced success or quality of offspring with advancing age of the mother (7). Advanced maternal age has known negative effects on offspring health, lifespan, and fertility in humans and other species (8–16). In many taxa, including rotifers, *Daphnia*, *Drosophila*, and soil mites, offspring from older mothers have shorter lives, lower reproductive success, or both (8, 11, 15–18). Field studies of several species of mammals and birds have shown that offspring with older parents exhibit lower survival and recruitment and increased rates of senescence (14, 19). In humans, advanced maternal age is associated with reduced lifespan (20) and health (9, 21). In *Caenorhabditis elegans*, *Daphnia*, and rotifers, advanced maternal age also increases offspring size, alters development time, and increases variability in gene expression (16, 22–24).

Maternal effect senescence remains an interesting problem in life history evolution. Producing high-quality offspring that live long and prosper should, all else being equal, provide a selective advantage. Thus, the reduced quality of the offspring of old mothers demands an evolutionary explanation. Hamilton (2) was the first to recognize that age-related senescence can be explained by the decline with age in selection gradients on age-specific mortality and fertility. Previous work by Medawar (1) and Williams (25) had not identified all of the components of those selection gradients (26).

A similar approach to maternal effect senescence would look for patterns of selection gradients, on mortality and fertility, as functions of maternal age. Doing so, however, requires a model that goes beyond that of Hamilton (2), whose results are based on a model in which individuals are classified only by age. The calculation of fitness and selection gradients relating to maternal effect senescence requires a multistate, age-by-stage demographic model, where the “stage” refers to maternal age.

Moorad and Nussey (7) recently analyzed one aspect of maternal effect senescence: the effect on neonatal survival. Their analysis focused on maternal effects mediated by social interactions between mothers and offspring. In contrast, we developed a more general multistate model that can incorporate maternal age effects on age-specific survival and fertility throughout the life cycle and with which we can easily calculate selection gradients on any of those rates as joint functions of age and maternal age. Our model does not require social interactions between mothers and offspring. The approach could be easily modified to address other maternal effects, such as inducible defenses (27–33) and effects from maternal diet and maternal stress, which influence offspring survival and fecundity (22). We analyzed the model to characterize selection gradients on survival and fertility as functions of age and maternal age and to quantify the fitness impact of maternal age effects throughout the life cycle. It is known that multistate models can fundamentally alter the patterns of age-specific selection gradients, and thus the evolution of senescence, in the more familiar age-classified models (34).

We applied the model to the monogonont rotifer, *Brachionus manjavacas*, an ecologically important, microscopic, invertebrate animal. *B. manjavacas* has several features which make it a useful system in which to investigate maternal effects on offspring performance. When reproducing asexually, all individuals within a population are female and genetically identical, eliminating variability due to genetic recombination and paternal effects. Asexual *B. manjavacas* females make a significant investment in individual offspring, producing 25 to 30 large daughters serially over a reproductive period of approximately 10 d. *Brachionus* rotifers have direct development, with no larval stage or metamorphosis, and exhibit no posthatching parental care. Thus, maternal care does not contribute to maternal age effects in this species. In this study, rotifers were individually housed and monitored, permitting frequent individual-level measurements of lifespan and fecundity with high replication. Although *Brachionus* rotifers have been used in previous studies of maternal effects (16, 22, 27, 35), the impacts of maternal age effects on population dynamics and evolutionary fitness have not been investigated in these species.

We begin by describing the demographic model, the experimental system, and our estimation of the survival and fertility parameters. The model provides estimates of fitness, stable population structure, and selection gradients on survival and fertility. We use life table response experiment (LTRE) methods (e.g., refs. 26 and 36) to decompose the impact of maternal effect senescence on fitness into contributions from age- and maternal-age-specific survival and fertility. We compare the results for models with and without maternal effect senescence in both high growth rate (laboratory) and low growth rate (simulated natural) environments. Our results reveal that maternal effect senescence decreases fitness; nevertheless, the decline of selection gradients with maternal age provides the opportunity for it to evolve.

## The Demographic Model

Our demographic model uses the general age-by-stage structured approach thoroughly described by Caswell et al. (37). If $n_{i,j}\left(t\right)$ is the number of individuals in maternal age class i and age class j at time t, then the composition of the population is given by a column vector $\tilde{n}\left(t\right)$ that collects maternal ages within age classes:

$$\tilde{n}\left(t\right)=\left(\begin{array}{c} \begin{array}{c} n_{1,1}\left(t\right)\\ \vdots \\ n_{s,1}\left(t\right) \end{array}\\ \vdots \\ \begin{array}{c} n_{1,\omega }\left(t\right)\\ \vdots \\ n_{s,\omega }\left(t\right) \end{array} \end{array}\right).$$ [1]

We use a projection interval of 1 d. In our data, no individual reproduced after 16 d, so we set both the maximum age (ω) and the maximum maternal age (s) to 16 d.

An individual with maternal age i and age j produces $f_{ij}$ daughters in 1 d and survives to age j+1 with probability $p_{ij}$. These vital rates are incorporated into a fertility matrix $\tilde{F}$ and a survival matrix $\tilde{U}$. The population projection matrix $\tilde{A}$, which projects the population vector from one day to the next, is the sum of $\tilde{F}$ and $\tilde{U}$, and the population dynamics are given by

$$\tilde{n}\left(t+1\right)=\left(\tilde{U}+\tilde{F}\right)\tilde{n}\left(t\right)=\tilde{A}\;\tilde{n}\left(t\right).$$ [2]

Caswell et al. (37) describe in detail the construction of $\tilde{U}$ and $\tilde{F}$ for general stage-by-age matrix models. The special case where stage is maternal age is described in *SI Appendix*. To duplicate the results of our analyses, the reader needs the following:

### The Matrix $\tilde{U}$.

This block matrix takes the form

$$\tilde{U}=\left(\begin{array}{ccccc} 0 & & & \\ U_{1} & 0 & & \\ & U_{2} & 0 & \\ & & \ddots & 0\\ & & & U_{\omega -1} & 0 \end{array}\right).$$ [3]

The $U_{j}$ are s×s matrices with survival probabilities on the diagonal and zeros elsewhere:

$$U_{j}=\left(\begin{array}{cccc} p_{1,j} & & \\ & p_{2,j} & \\ & & \ddots \\ & & & p_{s,j} \end{array}\right).$$ [4]

### The Matrix $\tilde{F}$.

This matrix has a block first row composed of the s×s blocks $F_{j}$:

$$\tilde{F}=\left(\begin{array}{cccc} F_{1} & F_{2} & \ldots & F_{\omega } \end{array}\right).$$ [5]

The block $F_{j}$ is a fertility matrix for all females in age class j. Because the offspring of a mother of age j have maternal age j, the matrix $F_{j}$ contains zeros everywhere except in the jth row, where the vector $f_{j}=\left(f_{1,j},f_{2,j},\ldots ,f_{s,j}\right)$ appears.

A population described by model [**2**] will converge to a stable structure $\tilde{w}$ and grow exponentially at the rate log⁡λ, where λ is the largest eigenvalue of the projection matrix $\tilde{A}$, and $\tilde{w}$ is the corresponding right eigenvector. We treat the intrinsic growth rate λ associated with a phenotype as a measure of the fitness of that phenotype (e.g., refs. 2, 38, and 39). The selection gradient on a trait that affects $\tilde{F}$ or $\tilde{U}$ (e.g., $p_{ij}$ or $f_{ij}$) is the partial derivative of λ with respect to that trait (39). Formulas for these selection gradients are given in *SI Appendix*.

## Model Parameterization

The fertilities $f_{ij}$ and survival probabilities $p_{ij}$ are the building blocks of model [**2**]. We parameterize the dependence of these quantities on age and maternal age as follows.

**Fig. 1.**
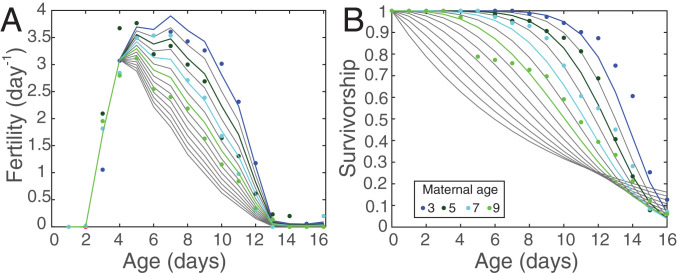
Fertility (*A*) and survival (*B*) schedules that underlie the projection matrix $\tilde{A}$. Observations are shown as solid circles, colored by their maternal age classification. Model fits to the data are shown as colored lines, and interpolated/extrapolated curves are shown in gray.

To describe the dependence of fertility on age and maternal age, we adapted the Coale–Trussell fertility model commonly used in human demography (40). In the model, fertility is the product of a “natural fertility” function that depends only on age and an additional factor that, after some fixed age, reduces the natural fertility. When fitted to our laboratory data using maternal age as the additional factor (as described in the *SI Appendix*), the Coale–Trussell model produces a fertility schedule that increases sharply between 2 d and 4 d of age, regardless of maternal age. The schedules for individuals with different maternal ages diverge after age 4 d. The fertility of individuals with older maternal age declines earlier and faster than the fertility of individuals with younger maternal age (Fig. 1*A*).

To describe the dependence of survival on age and maternal age, we write the survivorship function as a Weibull function, (e.g., ref. 41), with parameters that depend log-linearly on maternal age. The Weibull model is widely used and is sufficiently flexible to produce type I, II, or III survivorship schedules (*SI Appendix*). The resulting best-fit model predicts that survival probability decreases with maternal age for young individuals, but increases with maternal age for the very oldest individuals (Fig. 1*B*). These oldest individuals have extremely low fertility and are very rare in the population, so this old-age increase in survival with maternal age has negligible effect on our results.

### Model Modifications.

The projection matrix $\tilde{A}$ is estimated under laboratory culture conditions that lead to population growth rates that are unrealistically high for natural populations. This affects not only the growth rate, but also the population structure and selection gradients. To evaluate these effects, we created two hypothetical matrices describing stationary, rather than rapidly increasing populations. The first one, $\tilde{B}$, has reduced fertility, such as might result from resource limitation in nature. The fertilities in $\tilde{B}$ are obtained by dividing the fertilities in $\tilde{A}$ (all of which appear in the matrix $\tilde{F}$) by the net reproductive rate $R_{0}$; this yields a stationary population with an intrinsic growth rate of 1. The survival schedule and the shape and timing of reproduction are unchanged. The second hypothetical matrix, $\tilde{C}$, has increased mortality, such as might arise from predation or infection. To reduce λ to 1 for $\tilde{C}$, we imposed an additive mortality hazard to all age-specific mortality rates by multiplying the survival probabilities in $\tilde{A}$ (all of which appear in the matrix $\tilde{U}$) by a constant (0.3833).

The projection matrices $\tilde{A}$, $\tilde{B}$, and $\tilde{C}$ all include maternal effect senescence. To quantify the fitness costs of maternal effect senescence and to investigate the selective processes that could lead to its evolution, we compared each of these matrices with a hypothetical matrix in which offspring quality does not decline with increasing maternal age. We call these matrices ${\tilde{A}}^{\left(r\right)}$, ${\tilde{B}}^{\left(r\right)}$, and ${\tilde{C}}^{\left(r\right)}$, to indicate that maternal effect senescence has been removed. In these three matrices, all individuals, regardless of maternal age, have the fertility and survival schedules corresponding to a maternal age of 3 d in $\tilde{A}$, $\tilde{B}$, and $\tilde{C}$, respectively. This maternal age group has the highest fertility rates and the largest survival probabilities.

## Demographic and Evolutionary Analyses

The population projection matrices provide the link between the individual-level data from our laboratory experiments (16) and their ecological and evolutionary consequences for populations.

### Fitness and Population Structure.

The fitnesses of the six life histories described by each of the matrices are

$$\begin{array}{cc} \lambda \left(\tilde{A}\right)=1.967, & \lambda \left({\tilde{A}}^{\left(r\right)}\right)=1.975,\\ \lambda \left(\tilde{B}\right)=1.00, & \lambda \left({\tilde{B}}^{\left(r\right)}\right)=1.039,\\ \lambda \left(\tilde{C}\right)=1.00, & \lambda \left({\tilde{C}}^{\left(r\right)}\right)=1.0013. \end{array}$$ [6]

**Fig. 2.**
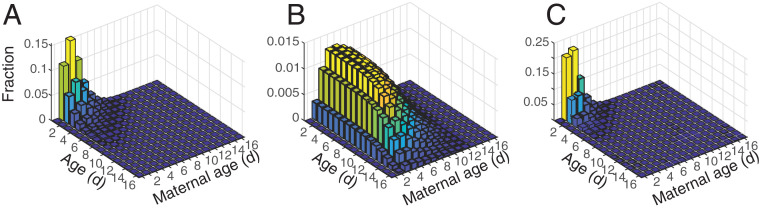
(*A*–*C*) Stable age-by-maternal-age distributions for a high-growth population $\tilde{A}$ (*A*), a low-fertility population $\tilde{B}$ (*B*), and a low-survival population $\tilde{C}$ (*C*), all with maternal effect senescence. The height of each bar, as well as its color, represents the portion of the stable population that is of that age and maternal age. Note that each panel has a different *z* scale and corresponding color scale.

In the high-growth, maternal-effect-senescent population, represented by $\tilde{A}$, the expected lifetime reproductive output ($R_{0}$) is 22.42. The stable population structure is dominated by young individuals born to young mothers (i.e., individuals of age 1 to 3 d and with a maternal age of 1 to 5 d); these individuals constitute 77% of the high-growth population (Fig. 2*A*). In the low-fertility, maternal-effect-senescent population, represented by $\tilde{B}$, we have adjusted the fertility so that λ=1 and $R_{0}=1$. As a result, the stable population structure is much flatter; young individuals of young mothers constitute only 11% of this population (Fig. 2*B*; note difference in scale). In the low-survival maternal-effect-senescent population, represented by $\tilde{C}$, $R_{0}$ and λ are also one. However, for this population, the stable population structure is even more skewed toward young ages and young maternal ages than that for $\tilde{A}$; young individuals born to young mothers constitute 90% of the population (Fig. 2*C*).

Removing maternal effect senescence increases λ (Eq. **6**), but the stable population structures are qualitatively unchanged (*SI Appendix*, Fig. S3). The fitness difference due to maternal effect senescence in high-growth laboratory conditions, as measured by the difference $\lambda \left(\tilde{A}\right)-\lambda \left({\tilde{A}}^{\left(r\right)}\right)$, is small (Δλ=−0.008). The same effect is present in the low-survival environment (Δλ=−0.0013). Under both high-growth-rate and low-survival conditions, almost all of the population is at young ages and young maternal ages, where the effect of maternal effect senescence is minimal. In the low-fertility population, the fitness difference $\lambda \left(\tilde{B}\right)-\lambda \left({\tilde{B}}^{\left(r\right)}\right)$ is larger than in the high-growth conditions (Δλ=−0.039).

### Selection Gradients and the Evolution of Maternal Effect Senescence.

The strength of selection is measured by the selection gradients, defined as the derivatives of λ with respect to survival or fertility (see the *SI Appendix* for their calculation). The potential for selection to lead to senescence varies directly with the rate at which the selection gradient declines with age (42).

**Fig. 3.**
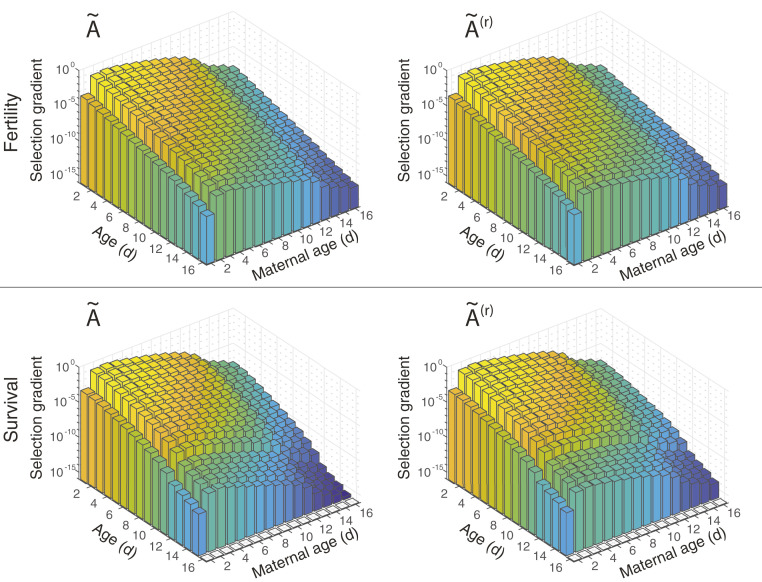
Selection gradients on survival and fertility in a high-growth environment, with and without the presence of maternal age effects. *Top* row shows the selection gradients on fertility, and *Bottom* row shows the selection gradients on survival. *Left* column shows the selection gradients with maternal effects, and *Right* column shows the selection gradients without maternal effects. Bars are colored by the magnitude of the selection gradient. Note that the *z* axis is log-scaled.

**Fig. 4.**
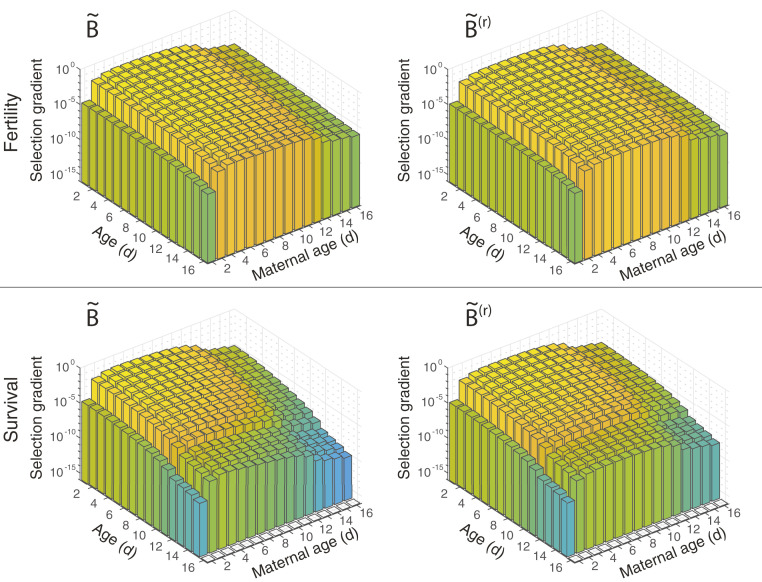
Selection gradients on survival and fertility in a low-fertility environment, with and without maternal age effects. *Top* row shows the selection gradients on fertility, and *Bottom* row shows the selection gradients on survival. *Left* column shows the selection gradients with maternal effects, and *Right* column shows the selection gradients without them. Bars are colored by the magnitude of the selection gradient. Note that the *z* axis is log-scaled.

The selection gradients on survival and fertility decline with age within any maternal age class (Figs. 3 and 4 and *SI Appendix*, Fig. S4), as they must since individuals cannot change their maternal age group. The differences in the selection gradients between young and old ages range from 1 to 10 orders of magnitude; the smallest differences occur under low-fertility conditions and the largest are in low-survival conditions. This decline provides the impetus for the evolution of age-related senescence. As expected (26), the selection gradients do not change qualitatively when growth rate is reduced.

The evolution of maternal effect senescence can be driven by a decline in selection gradients with increasing maternal age. We find that the selection gradients do, in fact, decline with maternal age, after maternal age 3 d for high-growth and low-survival populations (Fig. 3 and *SI Appendix*, Fig. S4) and after maternal age 4 or 5 d for low-fertility populations (Fig. 4). The declines in the selection gradients from their peaks are steep: 6 to 10 orders of magnitude in high-growth or low-survival conditions and 3 to 5 orders of magnitude in low-fertility conditions. That is, a unit reduction in survival or fertility at old maternal ages can be paid for by a much smaller increase, 1 hundredth to 1 millionth as large, at younger maternal ages.

This result implies that maternal effect senescence can evolve in the same way as age senescence. Traits that reduce survival or fertility of the offspring of older mothers can be balanced by much smaller improvements at younger maternal ages. Even in the absence of such pleiotropic effects, selection will be less efficient at removing deleterious mutations at older ages, permitting them to accumulate.

The matrices $\tilde{A}$, $\tilde{B}$, and $\tilde{C}$ already contain maternal effect senescence, and so the selection gradients calculated from them apply, strictly speaking, to increases in already existing maternal effects. In the life histories described by ${\tilde{A}}^{\left(r\right)}$, ${\tilde{B}}^{\left(r\right)}$, and ${\tilde{C}}^{\left(r\right)}$, maternal age effects have been removed. The selection gradients, on both survival and fertility, still decline with increasing maternal age, implying not only that selection favors maternal effect senescence when it is already present in *B. manjavacas*, but also that it can easily arise de novo.

### The Sources of Fitness Differences: LTRE Analysis.

The fitness difference due to maternal effect senescence is measured by the difference in λ between matrices with and without this maternal effect (e.g., between $\tilde{A}$ and ${\tilde{A}}^{\left(r\right)}$). The difference in λ, in turn, results from the differences in all of the vital rates at all combinations of age and maternal age. The contributions of each of these differences to the difference in fitness are calculated using an LTRE analysis (ref. 26, chap. 10). To focus on the contributions from the maternal age groups, and to separate those contributions into survival and fertility, we summed all of the contributions over age within each maternal age group.

**Fig. 5.**
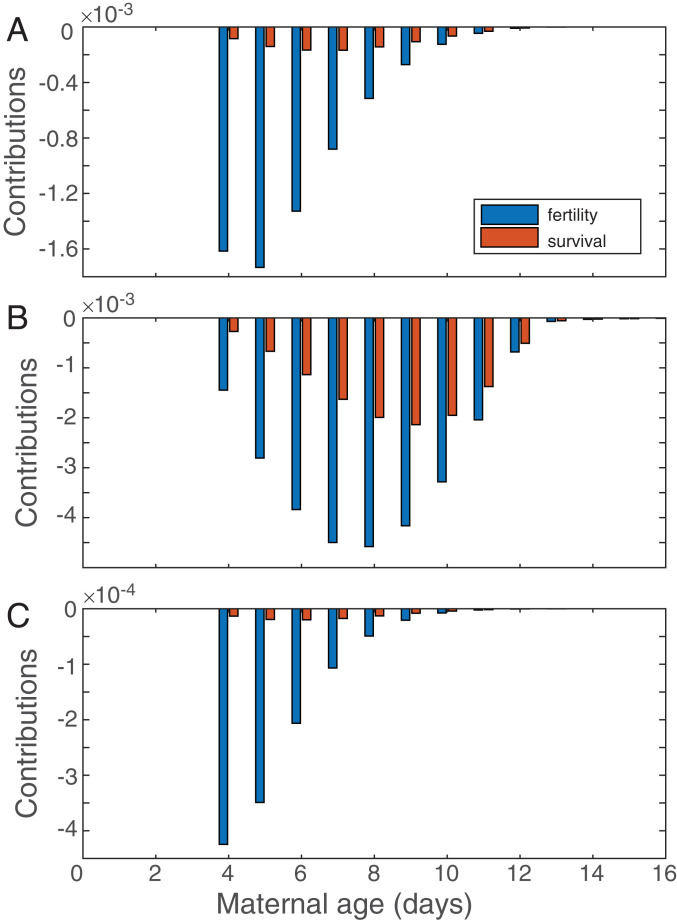
Results of the life table response experiments in the high-growth population (comparing $\tilde{A}$ with ${\tilde{A}}^{\left(r\right)}$) (*A*), the low-fertility population (comparing $\tilde{B}$ with ${\tilde{B}}^{\left(r\right)}$) (*B*), and the low-survival population (comparing $\tilde{C}$ with ${\tilde{C}}^{\left(r\right)}$) (*C*). The contributions of fertility and survival, summed across ages, show the effect on λ from changes in fertility and survival for offspring from a given maternal age. Note the change in scale between the panels.

Our analyses show that the decreased fitness resulting from maternal effect senescence is primarily caused by reduced lifetime fertility of offspring rather than reduced survival. Furthermore, the contributions from fertility reductions generally occur for younger maternal ages than do the contributions from survival reductions (Fig. 5).

The magnitude of the difference in fitness depends on environmental conditions. In particular, the difference is largest under low-fertility, moderate under high-growth, and smallest under low-survival conditions. In high-growth conditions, the largest contributions come from the decreases in fertility of individuals with maternal age between 4 and 6 d; the contributions from the decreases in survival peak at maternal ages 6 to 8 d (Fig. 5*A*). Under low-survival conditions, the largest fertility contributions are more concentrated at earlier maternal ages 4 to 5 d, and the contributions from survival are small (Fig. 5*C*).

Decreased survival due to maternal effect senescence contributes relatively more to the reduction in fitness in the low-fertility population than it does in either the high-growth or the low-survival populations. The largest contributions from fertility occur at maternal ages 7 to 9 d; the contributions from survival peak at maternal ages 8 to 10 d (Fig. 5*B*).

## Discussion

In this study, we used a multistate matrix population model to quantify the population and evolutionary consequences of maternal effect senescence in the rotifer *B. manjavacas*. We found that age-specific survival and fertility decrease with increasing maternal age and that this carries a cost in fitness compared to a hypothetical life cycle in which the maternal effect senescence has been removed. The fitness differences are primarily due to decreased fertility of individuals with early to intermediate maternal ages. The cost is obscured in luxurious (for a rotifer) laboratory conditions and in low-survival conditions, because these environments lead to population structures in which almost all individuals are young offspring of young mothers. The cost is larger in conditions characterized by reduced fertility. (Note that while density effects and ecological interactions might produce such stationary populations, all our models are linear and none include density dependence.)

The selection gradients we estimated are concave functions of maternal age and eventually decrease, by orders of magnitude, with increasing maternal age. These results imply that the two mechanisms by which senescence can evolve—antagonistic pleiotropy and mutation–selection balance—could also lead to the evolution of maternal effect senescence.

Moorad and Nussey (7) also found concave selection gradients in their analysis of neonatal survival. While our results complement theirs, our model differs. Their model includes quantitative genetic aspects to analyze maternal effects on neonatal survival resulting from social interactions between mothers and offspring. This approach deals with a single component of the life cycle and does not apply to the many species, including rotifers such as *B. manjavacas*, without intergenerational social interactions. In contrast, the experimental system and model framework that we developed allow us to incorporate maternal age effects throughout the life cycle of the offspring and to calculate selection gradients at every age and maternal age.

Our results are based on laboratory data for a single species, but are more generally applicable. The demographic structure of the model is unremarkable (Weibull survival and Coale–Trussell fertility) and likely to apply qualitatively to many species. The concave pattern of selection gradients, first increasing and eventually decreasing with increasing maternal age, is evident in both the high-growth ($\tilde{A}$) and low-growth ($\tilde{B}$ and $\tilde{C}$) populations. Thus, our findings are relevant to natural populations and are not artifacts of the laboratory environment. By removing maternal age effects from the matrices, we showed that the results are not caused by maternal effect senescence itself, suggesting that maternal effect senescence may evolve de novo.

The potential ease with which maternal effect senescence can arise raises the question of why it is not observed in all species. For example, the extremely diverse group of teleost fishes generally produces offspring of higher quality with advanced maternal age; older mothers produce more eggs and those eggs have a higher likelihood of hatching and of surviving to maturity (43, 44). In contrast to rotifers, fish exhibit indeterminate growth, increasing fertility schedules, and larger investment in offspring with increasing maternal age or size. Maternal effect senescence also appears to be absent in some species that exhibit postnatal maternal care. For example, in marmots, daughters from older mothers may have increased reproductive success, possibly due to improvements in care of offspring as mothers age (45).

The demographic model and analytical approach we developed require only measurements of survival and fertility as functions of age and maternal age. The maternal age classes need not correspond exactly to age classes (e.g., mothers could be grouped into “young” and “old”). With some extra effort, it is possible to extend the analysis to maternal age effects in stage-classified rather than age-classified life histories or to include positive as well as negative maternal age effects.

The mechanisms underlying the demographic effects of maternal age in *B. manjavacas* are unknown (16). Our matrix framework provides a way to incorporate effects of a variety of mechanisms, such as social interactions between mothers and offspring (7), maternal energy allocation changes (43, 44), and associated epigenetic effects (46, 47). Developing explicit mechanistic links is an interesting open problem.

The decline of selection gradients on survival and fertility with increasing age has long provided the framework for understanding age-related senescence. The decline of selection gradients with increasing maternal age does the same for maternal effect senescence. For a general class of life histories, the selection gradients are concave functions of maternal age, increasing at first, but eventually declining. In one specific case, based on high-resolution laboratory data, the declines are substantial, implying the possibility (with the same caveats involved in age-related senescence) for the evolution of maternal effect senescence. This framework unifies the demographic approaches to age-related and maternal effect senescence in the study of aging.

## Materials and Methods

### Notation.

Uppercase symbols in boldface type denote matrices (e.g., U), and lowercase symbols in boldface type (e.g., w) denote vectors. Symbols with a tilde (e.g., $\tilde{n}$) are block structured, with maternal ages grouped within ages.

### Life Table Experiments.

We conducted life table experiments using the Russian strain of the monogonont rotifer *B. manjavacas* (BmanRUS) as in a previously published study (16). We synchronized the maternal ages of the great-grandmaternal and grandmaternal generations for the experimental maternal (F0) cohort by collecting eggs from 3- to 5-d-old females for two generations to avoid undefined parental effects in the experimental populations. To obtain the F0, eggs from the age-synchronized grandmaternal culture were harvested by vortexing and hatched over 16 h. Neonates were deposited individually into 1 mL of 15 parts per thousand seawater and $6\times 10^{5}$ cells mL^−1^ of the chlorophyte algae, *Tetraselmis suecica*, in wells of 24-well tissue culture plates (*n* = 187). To obtain the F1 cohorts, at maternal ages of 3, 5, 7, and 9 d we isolated one female neonate hatched within the previous 24 h per F0 female. F1s were placed individually in wells of 24-well plates with seawater and algae as above (*n* = 72 for each F1 cohort). Every 24 h, we recorded survival and the number of live offspring for each F0 and F1 individual; the female was then transferred to a new well with fresh algae and seawater. Survivorship data were right censored in cases where individuals were lost prior to death.

### Data Availability.

Life table data as well as the ${\tilde{U}}_{i}$ and ${\tilde{F}}_{i}$ matrices are available in the *SI Appendix*. Additional data files related to this paper are available in GitHub at https://github.com/chrissy3815/rotifer-moms/tree/master.

## Supplementary Material

Supplementary File

## References

[1] Medawar P. B., An Unsolved Problem in Biology (H. K. Lewis, London, England, 1952).

[2] Hamilton W. D., The moulding of senescence by natural selection. J. Theor. Biol. 12, 12–45 (1966). 6015424 10.1016/0022-5193(66)90184-6

[3] Fabian D., Flatt T., The evolution of aging. Nat. Educ. Knowl. 3, 9 (2011).

[4] Rose M. R., Evolutionary Biology of Aging (Oxford University Press, Oxford, UK, 1990).

[5] Jones O. R., Diversity of ageing across the tree of life. Nature 505, 169–173 (2014). 24317695 10.1038/nature12789 PMC4157354

[6] Shefferson R. P., Salguero-Gómez R., Jones O. R., Eds., The Evolution of Senescence in the Tree of Life (Cambridge University Press, 2017).

[7] Moorad J. A., Nussey D. H., Evolution of maternal effect senescence. Proc. Natl. Acad. Sci. U.S.A. 113, 362–367 (2016). 26715745 10.1073/pnas.1520494113 PMC4720302

[8] Lansing A. I., A transmissible, cumulative and reversible factor in aging. J. Gerontol. 2, 228–239 (1947). 20265000 10.1093/geronj/2.3.228

[9] de la Fuente-Fernandez R., Maternal effect on Parkinson’s disease. Ann. Neurol. 48, 782–787 (2000). 11079542 10.1002/1531-8249(200011)48:5<782::aid-ana12>3.3.co;2-5

[10] Hercus M. J., Hoffman A. A., Maternal and grandmaternal age influence offspring fitness in *Drosophila*. Proc. R. Soc. Lond. B 267, 2105–2110 (2000). 10.1098/rspb.2000.1256 PMC1690784 11416916

[11] Fox C. W., Bush M. L., Wallin W. G., Maternal age affects offspring lifespan of the seed beetle, *Callosobruchus maculatus*. Funct. Ecol. 17, 811–820 (2003).

[12] Talge N. M., Neal C., Glover V., The early stress, translational research and prevention science network: Fetal and neonatal experience on child and adolescent mental health, antenatal maternal stress and long-term effects on child neurodevelopment: How and why? J. Child Psychol. Psychiatry 48, 246–261 (2007). 10.1111/j.1469-7610.2006.01714.x PMC11016282 17355398

[13] Bouwhuis S., Vedder O., Becker P. H., Sex-specific pathways of parental age effects on offspring lifetime reproductive success in a long-lived seabird. Evolution 69, 1760–1771 (2015). 26095174 10.1111/evo.12692

[14] Schroeder J., Nakagawa S., Rees M., Mannarelli M. E., Burke T., Reduced fitness in progeny from old parents in a natural population. Proc. Natl. Acad. Sci. U.S.A. 112, 4021–4025 (2015). 25775600 10.1073/pnas.1422715112 PMC4386340

[15] Bloch Qazi M. C., Transgenerational effects of maternal and grandmaternal age on offspring viability and performance in *Drosophila melanogaster*. J. Insect Physiol. 100, 43–52 (2017). 28529156 10.1016/j.jinsphys.2017.05.007

[16] Bock M. J., Jarvis G. C., Corey E. L., Stone E. E., Gribble K. E., Maternal age alters offspring lifespan, fitness, and lifespan extension under caloric restriction. Sci. Rep. 9, 3138 (2019). 30816287 10.1038/s41598-019-40011-z PMC6395700

[17] Kern S., Ackerman M., Stearns S. C., Kawecki T. J., Decline in offspring viability as a manifestation of aging in *Drosophila melanogaster*. Evolution 55, 1822–1831 (2001). 11681737 10.1111/j.0014-3820.2001.tb00831.x

[18] Benton T. G., St. Clair J. J. H., Plaistow S. J., Maternal effects mediated by maternal age: From life histories to population dynamics. J. Anim. Ecol. 77, 1038–1046 (2008). 18631260 10.1111/j.1365-2656.2008.01434.x

[19] Roach D. A., Carey J. R., Population biology of aging in the wild. Annu. Rev. Ecol. Evol. Syst. 45, 421–443 (2014).

[20] Bell A. G., The Duration of Life and Conditions Associated with Longevity: Study of the Hyde Genealogy (Genealogical Record Office, Washington, DC, 1918).

[21] Farrer L., Cupples A., Kiely D. K., Conneally P. M., Myers R. H., Inverse relationship between age at onset of Huntington disease and paternal age suggests involvement of genetic imprinting. Am. J. Hum. Genet. 50, 528–535 (1992). 1531729 PMC1684271

[22] Gribble K. E., Jarvis G., Bock M., Mark Welch D. B., Maternal caloric restriction partially rescues the deleterious effects of advanced maternal age on offspring. Aging Cell 13, 623–630 (2014). 24661622 10.1111/acel.12217 PMC4116445

[23] Plaistow S. J., Shirley C., Collin H., Cornell S. J., Harney E. D., Offspring provisioning explains clone-specific maternal age effects on life history and life span in the water flea, *Daphnia pulex*. Am. Nat. 186, 376–389 (2015). 26655355 10.1086/682277

[24] Perez M. F., Lehner B., Intergenerational and transgenerational epigenetic inheritance in animals. Nat. Cell Biol. 21, 143–151 (2019). 30602724 10.1038/s41556-018-0242-9

[25] Williams G. C., Pleiotropy, natural selection, and the evolution of senescence. Evolution 11, 398–411 (1957).

[26] Caswell H., Matrix Population Models: Construction, Analysis, and Interpretation (Sinauer Associates, 2001).

[27] Gilbert J. J., Rotifer ecology and embryological induction. Science 151, 1234–1237 (1966). 5910006 10.1126/science.151.3715.1234

[28] Gilbert J. J., *Asplancha* and postero-lateral spine induction in *Brachionus calyciflorus*. Arch. Hydrobiol. 64, 1–62 (1967).

[29] Krueger D. A., Dodson S. I., Embryological induction and predation ecology in *Daphnia pulex*. Limnol. Oceanogr. 26, 219–223 (1981).

[30] Havel J. E., Dodson S. I., *Chaoborus* predation on typical and spined morphs of *Daphnia pulex*: Behavioral observations. Limnol. Oceanogr. 29, 487–494 (1984).

[31] Gilbert J. J., Susceptibilities of ten rotifer species to interference from *Daphnia pulex*. Ecology 69, 1826–1838 (1988).

[32] Gilbert J. J., “Kairomone-induced morphological defenses in rotifers” in The Ecology and Evolution of Inducible Defenses, Tollrian R., Harvell C. D., Eds. (Princeton University Press, Princeton, NJ, 1999), pp. 127–141.

[33] Gilbert J. J., Specificity of crowding response that induces sexuality in the rotifer *Brachionus*. Limnol. Oceanogr. 48, 1297–1303 (2003).

[34] Caswell H., Salguero-Gómez R., Age, stage and senescence in plants. J. Ecol. 101, 585–595 (2013). 23741075 10.1111/1365-2745.12088 PMC3664411

[35] Kaneko G., Calorie restriction-induced maternal longevity is transmitted to their daughters in a rotifer. Funct. Ecol. 25, 209–216 (2011).

[36] Caswell H., Sensitivity Analysis: Matrix Methods in Demography and Ecology (Springer, 2019).

[37] Caswell H., de Vries C., Hartemink N., Roth G., van Daalen S. F., Age × stage-classified demographic analysis: A comprehensive approach. Ecol. Monogr. 88, 560–584 (2018). 30555177 10.1002/ecm.1306 PMC6283253

[38] Metz J. A. J., Nisbet R. M., Geritz S. A. H., How should we define ‘fitness’ for general ecological scenarios? Trends Ecol. Evol. 7, 198–202 (1992). 21236007 10.1016/0169-5347(92)90073-K

[39] Lande R., A quantitative genetic theory of life history evolution. Ecology 63, 607–615 (1982).

[40] Coale A. J., Trussell T. J., Model fertility schedules: Variations in the age structure of childbearing in human populations. Popul. Index 40, 185–258 (1974). 12333625

[41] Cox D., Oakes D., Analysis of Survival Data (Monographs on Statistics and Applied Probability, Chapman & Hall, London, UK, 1984), vol. 21.

[42] Caswell H., Shyu E., “Senescence, selection gradients and mortality” in The Evolution of Senescence in the Tree of Life, Shefferson R. P., Jones O. R., Salguero-Gómez R., Eds. (Cambridge University Press, 2017), pp. 56–82.

[43] Green B. S., Maternal effects in fish populations. Adv. Mar. Biol. 54, 1–105 (2008). 18929063 10.1016/S0065-2881(08)00001-1

[44] Marshall D. J., Allen R. M., Crean A. J., “The ecological and evolutionary importance of maternal effects in the sea” in Oceanography and Marine Biology: An Annual Review, Gibson R. N., Atkinson R. J. A., Gordon J. D. M., Eds. (Taylor & Francis, 2008), vol. 46, pp. 203–250.

[45] Kroeger S. B., Blumstein D. T., Armitage K. B., Reid J. M., Martin J. G., Older mothers produce more successful daughters. Proc. Natl. Acad. Sci. U.S.A. 117, 4809–4814 (2020). 32071200 10.1073/pnas.1908551117 PMC7060700

[46] Moore A. M., Persistent epigenetic changes in adult daughters of older mothers. Epigenetics 14, 467–476 (2019). 30879397 10.1080/15592294.2019.1595299 PMC6557560

[47] Markunas C. A., Maternal age at delivery is associated with an epigenetic signature in both newborns and adults. PLoS One 11, 1–11 (2016). 10.1371/journal.pone.0156361 PMC4934688 27383059

