# Supplementary material for: A demographic and evolutionary analysis of maternal effect senescence
Source: Proc Natl Acad Sci U S A. 2020 Jun 29;117(28):16431–7. doi: 10.1073/pnas.1919988117 (PMC7368264; doi:10.1073/pnas.1919988117)
Supplement: Supplementary File [file pnas.1919988117.sapp.pdf]

PNAS
www.pnas.org

Supplementary Information for

A Demographic and Evolutionary Analysis of Maternal Effect Senescence

Christina M. Hernández, Silke F. van Daalen, Hal Caswell, Michael G. Neubert, and Kristin E. Gribble

Kristin E. Gribble.
E-mail: kgribble@mbl.edu

This PDF file includes:

Supplementary text
Figs. S1 to S4
SI References

www.pnas.org/cgi/doi/10.1073/pnas.1919988117

Supporting Information Text

A. Fertility model: Coale and Trussell. The Coale and Trussell model (1), from human demography, represents differences in the fertility schedules of populations by using a 'natural fertility schedule' ($\tilde{m}(a)$), an age at which the population-specific schedules can diverge from the natural fertility schedule (a_0), and a parameter that controls how rapidly the schedules diverge (θ). The model is

$$m(a) = \tilde{m}(a) \exp[-v(a)], \tag{1}$$

$$v(a) = \begin{cases} 0 & a \leq a_0 \\ \theta(a - a_0) & a > a_0. \end{cases} \tag{2}$$

The Coale and Trussell model is typically fit to each population separately by choosing θ and a_0 to satisfy some statistical criterion (e.g., minimizing the sum of squared deviations between the model and the data).

If we had observed the fertility schedules of individuals in every maternal age class, we could have used model [1]–[2] directly. We, however, need to estimate fertility schedules for maternal ages that we did not measure in the laboratory experiment by interpolating among those we did. We therefore amended the Coale and Trussell model to incorporate the effects of maternal age g:

$$m(a, g) = \tilde{m}(a) \exp[-v(a, g)], \tag{3}$$

$$v(a, g) = \begin{cases} 0 & a \leq a_0 \\ \theta \cdot (g - \tilde{g}) \cdot (a - a_0) & a > a_0. \end{cases} \tag{4}$$

We now imagine that the "natural fertility schedule" is associated with a "natural maternal age" which, in model [3]–[4], is the new parameter $\tilde{g}$.

To fit model [3]–[4], we minimized the sum of squared deviations between the model and the data over all of the possible choices of

$\tilde{\mathbf{m}}(\mathbf{a})$. We assumed that natural fertility schedule $\tilde{m}(a)$ was one of five functions: one of the four observed fertility schedules for mothers with maternal age equal to 3, 5, 7 or 9; or the average fertility schedule over all maternal ages.

$\tilde{\mathbf{g}}$. With each of the five candidate natural fertility schedules, we associate exactly one value of $\tilde{g}$. For the four observed schedules, with maternal ages 3, 5, 7, and 9, we set $\tilde{g}$ equal to the maternal age. For the fifth, average, candidate schedule we set $\tilde{g} = 6$.

$\mathbf{a_0}$. Based on visual inspection of the observed fertility schedules (Fig. 1), we assumed that $a_0 \in \{2, 3, 4, 5\}$.

θ. The rate parameter θ was assumed to be a real positive number.

The best fit is achieved by the 'average' fertility schedule for $\tilde{m}(x)$, with $\tilde{g} = 6$, $a_0 = 4$, and $\theta = 0.0192$. The data, model fits, and extrapolated curves are shown in the main text (Fig. 1A).

B. Mortality model: Weibull. The Weibull model is a common representation of the time-to-failure in survival probability estimation for living organisms. It is a continuous probability distribution described by two parameters, and the shape of the probability density function (*pdf*) is very flexible. From the individual culture experiments described in (2), we have the time-to-death of many individual rotifers—with maternal ages 3, 5, 7, and 9 days—and we used this data to fit the Weibull distribution:

$$f(x|a, b) = \frac{a}{b} \left(\frac{x}{a}\right)^{b-1} e^{(-x/a)^b}. \tag{5}$$

The failure time, x, of each rotifer in the dataset is the oldest age (days) at which it was observed alive. The Weibull *pdf* [5] is fit to observed failure times in each maternal age cohort, yielding four Weibull models. Inspection of the parameters from those four models indicates that the parameters a and b vary log-linearly with maternal age group (Fig. S1), such that the Weibull parameters are themselves parametric functions of maternal age group, g. Although a linear model would fit equally well to the four points (Fig. S1), the log-linear form is necessary so that the Weibull parameters will both be non-negative for maternal age groups up to $g = 16$. With this formulation, the time-to-death description of mortality probabilities is a function of a single variable, g, which varies among groups, and four parameters that are defined for the population as a whole (the slope and intercept of the log-linear functions for a and b). The resulting Weibull *pdf*s are shifted towards earlier and more evenly spread times of death as g increases (Fig. S2). The resulting survivorship curves are shown in the main text (Fig. 1B).

C. Birthflow model: fertility and survivorship curves. Birth and death for the lab-cultured rotifers are continuous processes, and by censusing once per day, information on the age of an individual is limited. Neonates were assigned an age of 0 on the day that they were first counted in the census, but their true ages were between 0 and 1. Therefore, estimates of both fecundity and survival probability represent averages over the census interval (1 day), and a birth-flow model (3) must be used to build up the transition matrix, $\tilde{\mathbf{A}}$.

The survivorship curve (the proportion of neonates that survive to each age) was calculated using $1 - \mathcal{F}(x|a,b)$, where $\mathcal{F}$ is the cumulative distribution function for the Weibull model [5]. The transition probabilities for the matrix model formulation are given by

$$P_i = \frac{l_i + l_{i+1}}{l_{i-1} + l_i}, \qquad [6]$$

where P_i is the probability of surviving from age class i to age class $i+1$, and l_i, the probability of surviving to age i, is the survivorship curve derived from the Weibull model [5].

Likewise, the observed number of offspring produced by an individual in an interval averages over several processes that are unobserved during the interval. Production of neonates by a given age class, F_i,

$$F_i = \frac{l_0 + l_1}{2} \cdot \frac{m_i + P_i \cdot m_i + 1}{2}, \qquad [7]$$

is determined by the fertility at that age, the fertility at the next age, the probability that individuals survive to that next age, and the probability that the offspring survive long enough to be counted. The average offspring must survive 0.5 days to be counted, and the fertility of the mother is an average, weighted by the probability of surviving to the next age (3). The values of the m_i in equation [7] come directly from the fertility model described in Section A.

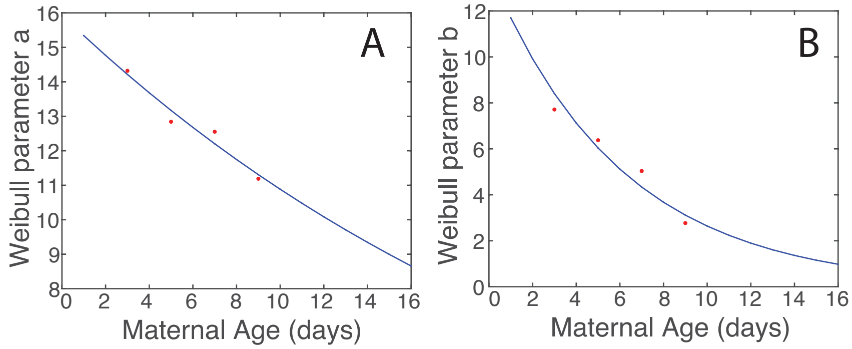

Fig. S1. Weibull model parameters a (panel A) and b (panel B) vary log-linearly with maternal age group. Red points represent the observed Weibull parameters for maternal ages 3, 5, 7, and 9. The blue lines are the log-linear best fits, $a = \exp(-0.038 \cdot g + 2.77)$ and $b = \exp(-0.165 \cdot g + 2.63)$.

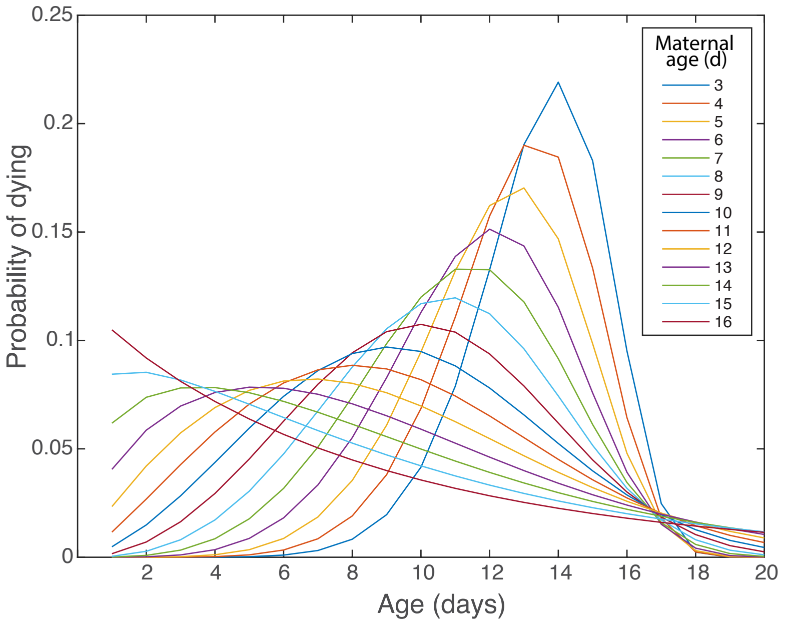

Fig. S2. Weibull probability density functions from the full model that varies with g, for $g \in [3, 4, \ldots, 16]$. The blue curve with the highest peak is for maternal age group 3, and the red curve that is highest at age 1 day is for maternal age group 16.

D. Multistate model construction. In what follows, "stage" refers to maternal age class. The matrix $\mathcal{D}(\mathbf{x})$ contains $\mathbf{x}$ on the diagonal and zeros elsewhere. The transpose of $\mathbf{x}$ is $\mathbf{x}^{\mathsf{T}}$. The vector $\mathbf{e}_j$ is the jth unit vector: a vector with a 1 in the jth entry and zeros elsewhere. The symbol $\otimes$ denotes the Kronecker product. The vec operator turns a $m \times n$ matrix into a $mn \times 1$ vector by stacking the columns. Subscripts are used to denote the dimension of matrices and vectors; $\mathbf{0}_{m\times n}$ is a $m \times n$ matrix of zeros, and $\mathbf{1}_m$ is a $m \times 1$ vector of ones.

The details of construction of age-stage models, using the vec-permutation formulation, are given in (4). In our particular case of maternal age effects, we begin with the vectors $\mathbf{p}_j$, containing stage-specific survival probabilities at age j and $\mathbf{f}_j$ containing stage-specific fertilities at age j. Define the matrices

$$\mathbf{U}_j = \mathcal{D}(\mathbf{p}_j), \quad \text{for} \quad j = 1, \ldots, \omega, \qquad [8]$$

and

$$\mathbf{D}_i = \text{subdiag}(\mathbf{1}_{\omega-1}), \quad \text{for} \quad i = 1, \ldots, s. \qquad [9]$$

Let $\mathbb{U}$ and $\mathbb{D}$ be block diagonal matrices with the $\mathbf{U}_j$ and $\mathbf{D}_i$ on the diagonal, and let $\mathbf{K}$ be the vec-permutation matrix. Then

$$\tilde{\mathbf{U}} = \mathbf{K}^{\mathsf{T}}\, \mathbb{D}\, \mathbf{K}\, \mathbb{U}. \qquad [10]$$

For fertility, define the fertility matrices

$$\mathbf{F}_j = \mathbf{e}_j \mathbf{f}_j^{\mathsf{T}}, \qquad j = 1, \ldots, \omega \qquad [11]$$

and the $\omega \times \omega$ age assignment matrices $\mathbf{H}_i$, which are all identical, with ones in the first row and zeros elsewhere. Create the block diagonal matrices $\mathbb{F}$ and $\mathbb{H}$. Then

$$\tilde{\mathbf{F}} = \mathbf{K}^{\mathsf{T}}\, \mathbb{H}\, \mathbf{K}\, \mathbb{F}. \qquad [12]$$

E. Selection gradients and LTRE calculations. The selection gradients on the stage-specific survival probability at age j are given by

$$\frac{\partial \lambda}{\partial \mathbf{p}_j^{\mathsf{T}}} = \frac{\partial \lambda}{\partial \text{vec}^{\mathsf{T}} \tilde{\mathbf{U}}} \frac{\partial \text{vec}\, \tilde{\mathbf{U}}}{\partial \text{vec}^{\mathsf{T}} \mathbf{U}_j} \frac{d\text{vec}\, \mathbf{U}_j}{d\mathbf{p}_j^{\mathsf{T}}}. \qquad [13]$$

Without loss of generality, assume that the eigenvectors of $\tilde{\mathbf{A}}$ are scaled so that $\tilde{\mathbf{w}}^{\mathsf{T}}\tilde{\mathbf{v}} = 1$. Then the first term in [13] is

$$\frac{\partial \lambda}{\partial \text{vec}^{\mathsf{T}} \tilde{\mathbf{U}}} = \tilde{\mathbf{w}}^{\mathsf{T}} \otimes \tilde{\mathbf{v}}^{\mathsf{T}}. \qquad [14]$$

The third term is

$$\frac{d\text{vec}\, \mathbf{U}_j}{d\mathbf{p}_j^{\mathsf{T}}} = \mathcal{D}\left(\text{vec}\, \mathbf{I}_s\right) \left(\mathbf{I}_s \otimes \mathbf{1}_s\right). \qquad [15]$$

The second term can be expressed in terms of two matrices (5)

$$\mathbf{Q}_i = \begin{pmatrix} \mathbf{0}_{s\times(i-1)s} & \mathbf{I}_s & \mathbf{0}_{s\times(\omega-i)} \end{pmatrix} \qquad [16]$$

and

$$\mathbf{R}_i = \begin{pmatrix} \mathbf{0}_{(i-1)s\times s} \\ \mathbf{I}_s \\ \mathbf{0}_{(\omega-i)s\times s} \end{pmatrix}. \qquad [17]$$

In terms of these matrices,

$$\frac{\partial \text{vec}\, \tilde{\mathbf{U}}}{\partial \text{vec}^{\mathsf{T}} \mathbf{U}_j} = \mathbf{Q}_{j-1} \otimes \mathbf{R}_j \qquad [18]$$

(see (5) for proof).

The selection gradients on stage-specific fertility at age j are

$$\frac{\partial \lambda}{\partial \mathbf{f}_j^{\mathsf{T}}} = \frac{\partial \lambda}{\partial \text{vec}^{\mathsf{T}} \tilde{\mathbf{F}}} \frac{\partial \text{vec}\, \tilde{\mathbf{F}}}{\partial \text{vec}^{\mathsf{T}} \mathbf{F}_j} \frac{d\text{vec}\, \mathbf{F}_j}{d\mathbf{f}_j^{\mathsf{T}}}, \qquad [19]$$

the terms of which are

$$\frac{\partial \lambda}{\partial \text{vec}^{\mathsf{T}} \tilde{\mathbf{F}}} = \tilde{\mathbf{w}}^{\mathsf{T}} \otimes \tilde{\mathbf{v}}^{\mathsf{T}}, \qquad [20]$$

$$\frac{\partial \text{vec}\, \tilde{\mathbf{F}}}{\partial \text{vec}^{\mathsf{T}} \mathbf{F}_j} = \mathbf{Q}_j \otimes \mathbf{R}_1, \text{ and} \qquad [21]$$

$$\frac{d\text{vec}\, \mathbf{F}_j}{d\mathbf{f}_j^{\mathsf{T}}} = \mathbf{I}_s \otimes \mathbf{e}_j. \qquad [22]$$

The derivatives in expressions [13] and [19] provide the material for LTRE decomposition of the differences in λ between the life histories with and without maternal age senescence. See (3, Chap. 10) for a general discussion of LTRE calculations and (6) for a presentation in terms of matrix calculus. We use the latter approach here.

We use $\tilde{\mathbf{A}}^{(r)}$, $\tilde{\mathbf{B}}^{(r)}$, and $\tilde{\mathbf{C}}^{(r)}$ as reference matrices. The difference $\Delta\lambda$ between the original matrix and the reference matrix is, to first order,

$$\Delta\lambda \approx \sum_{j=1}^{\omega} \frac{\partial\lambda}{\partial p_j^{\mathsf{T}}} \Delta\mathbf{p}_j + \sum_{j=1}^{\omega} \frac{\partial\lambda}{\partial f_j^{\mathsf{T}}} \Delta\mathbf{f}_j, \qquad [23]$$

where the derivatives are evaluated midway between the two survival and fertility schedules.

Define contribution matrices $\mathbf{C}_p$ and $\mathbf{C}_f$, where the ith row of $\mathbf{C}_p$, which we write as $\mathbf{C}_p(i,:)$, contains the contributions of differences in stage-specific survival at age i to $\Delta\lambda$, and similarly for $\mathbf{C}_f(i,:)$. These matrices are given by

$$\mathbf{C}_p(i,:) = \frac{\partial\lambda}{\partial \mathbf{p}_i^{\mathsf{T}}} \mathcal{D}(\Delta\mathbf{p}_i), \qquad i = 1,\ldots,\omega, \qquad [24]$$

$$\mathbf{C}_f(i,:) = \frac{\partial\lambda}{\partial \mathbf{f}_i^{\mathsf{T}}} \mathcal{D}(\Delta\mathbf{f}_i), \qquad i = 1,\ldots,\omega. \qquad [25]$$

The contributions shown in Fig. 5 of the main text are obtained by summing over ages; i.e., as column sums of $\mathbf{C}_p$ and $\mathbf{C}_f$.

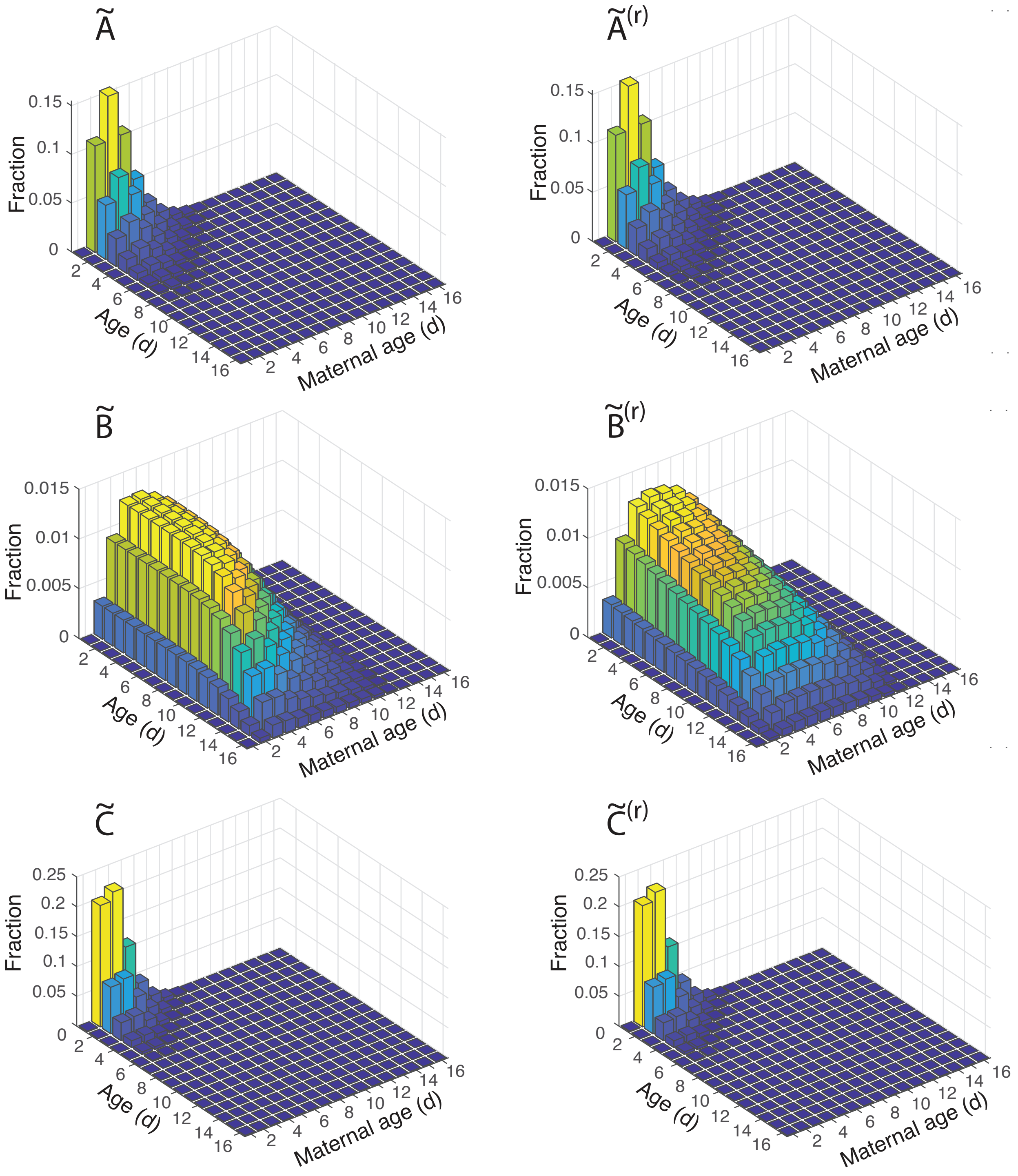

Fig. S3. Stable age-by-maternal-age distributions for all six population models: high-growth population with maternal age effects ($\tilde{\mathbf{A}}$, top left), high-growth population without maternal age effects ($\tilde{\mathbf{A}}^{(r)}$, top right), fertility-adjusted low-growth population with maternal age effects ($\tilde{\mathbf{B}}$, center left), fertility-adjusted low-growth population without maternal age effects ($\tilde{\mathbf{B}}^{(r)}$, center right), survival-adjusted low-growth population with maternal effects ($\tilde{\mathbf{C}}$, bottom left), and survival-adjusted low-growth population without maternal effects ($\tilde{\mathbf{C}}^{(r)}$, bottom right). The height of each bar represents the percent of the stable population that is of that age and maternal age. The bars are colored by their height, corresponding with the values on the z-axis. The colorbars and z-axis are consistent within each row of sub-panels ($\tilde{\mathbf{A}}$ and $\tilde{\mathbf{A}}^{(r)}$, $\tilde{\mathbf{B}}$ and $\tilde{\mathbf{B}}^{(r)}$, $\tilde{\mathbf{C}}$ and $\tilde{\mathbf{C}}^{r}$).

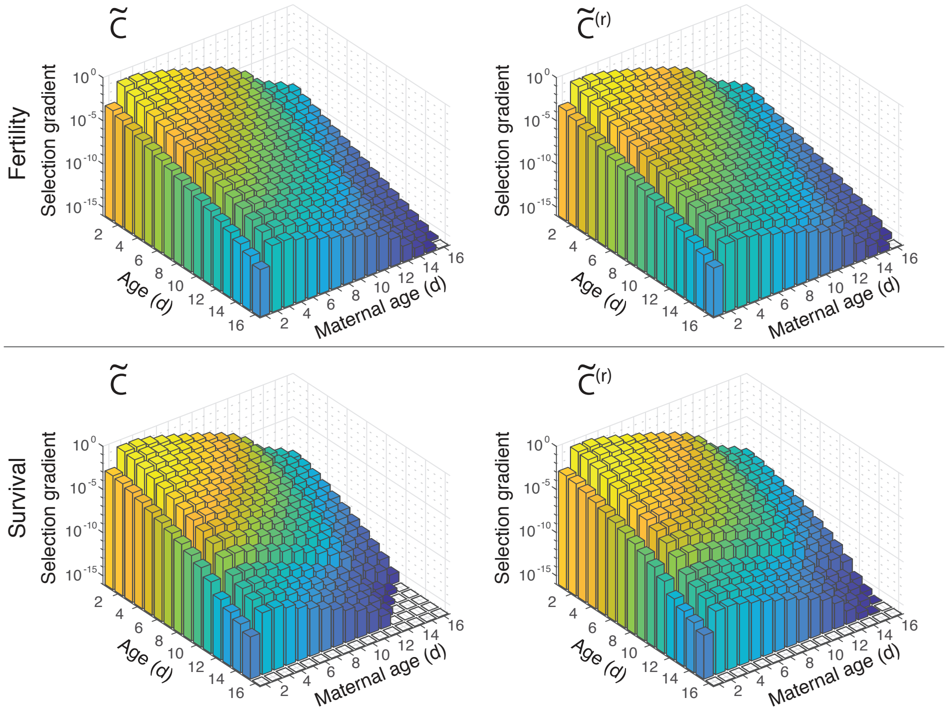

Fig. S4. Selection gradients on survival and fertility in low-growth life histories generated by adjusting survival, with ($\tilde{\mathbf{C}}$) and without ($\tilde{\mathbf{C}}^{(r)}$) the presence of maternal age effects. The panels in the top row are the selection gradients on fertility parameters, and the bottom row are for survival parameters. The left column shows the selection gradients with maternal effects, and the right column shows the selection gradients without maternal effects. The height of the bars is on a log-scale, and the bars are colored by their height, corresponding to the z-axis. All subpanels have the same z-axis limits and corresponding color axis.

References

1. AJ Coale, TJ Trussell, Model fertility schedules: variations in the age structure of childbearing in human populations. *Popul. Index*, 185–258 (1974).
2. MJ Bock, GC Jarvis, EL Corey, EE Stone, KE Gribble, Maternal age alters offspring lifespan, fitness, and lifespan extension under caloric restriction. *Sci. Reports* **9** (2019).
3. H Caswell, *Matrix Population Models: Construction, Analysis, and Interpretation.* (Sinauer Associates), (2001).
4. H Caswell, C de Vries, N Hartemink, G Roth, SF van Daalen, Age × stage-classified demographic analysis: a comprehensive approach. *Ecol. Monogr.* **88**, 560–584 (2018).
5. H Caswell, SF van Daalen, A note on the vec operator applied to unbalanced block-structured matrices. *J. Appl. Math.* **2016**, 1–3 (2016).
6. H Caswell, *Sensitivity Analysis: Matrix Methods in Demography and Ecology.* (Springer), (2019).
